# Smoking, alcohol, and dietary choices: evidence from the Portuguese National Health Survey

**DOI:** 10.1186/1471-2458-7-138

**Published:** 2007-07-03

**Authors:** Patrícia Padrão, Nuno Lunet, Ana Cristina Santos, Henrique Barros

**Affiliations:** 1 Department of Hygiene and Epidemiology, University of Porto Medical School, Alameda Prof. Hernâni Monteiro, 4200-319 Porto, Portugal; 2 Faculty of Nutrition and Food Sciences, University of Porto, Rua Dr. Roberto Frias, 4200-465 Porto, Portugal

## Abstract

**Background:**

Unhealthy lifestyle choices tend to cluster, but controversy remains regarding relationships between smoking and dietary habits. The aim of this study was to compare dietary intake and alcohol consumption, according to smoking status, in the Portuguese population.

**Methods:**

The study sample included all participants in the third Portuguese National Health Survey who were older than 19 years (20,302 women and 17,923 men).

Participants were selected from households in the five regions of Portugal (NUTS II classification), using a multi-stage random probability design. Trained interviewers conducted face-to-face interviews in each household and obtained information on social and demographic characteristics, lifestyle and health, smoking, and intakes of selected food and beverages. Age-adjusted and education-adjusted binomial and multinomial logistic regression models were fitted separately for males and females, to estimate the magnitude of the association between smoking and the consumption of various food and beverage groups.

**Results:**

When heavy smokers were compared with non-smokers, the odds ratio (OR) favouring soup consumption was 0.60 (95% Confidence Interval [95%CI]: 0.54–0.68) in males and 0.46 (95% CI: 0.33–0.65) in females. Similar ORs were observed for vegetables (males: OR = 0.56, 95%CI: 0.49–0.64; females: OR = 0.47, 95%CI: 0.32–0.69) and fruit (males: OR = 0.36, 95%CI: 0.31–0.41; females: OR = 0.29, 95%CI: 0.19–0.44). Overall, these food items were consumed at significantly lower levels as cigarette consumption increased. Heavy male smokers, compared to non-smokers, presented lower odds favouring milk consumption (OR = 0.89; 95%CI: 0.67–0.89). When heavy smokers were compared with non-smokers, the ORs favouring wine drinking, among heavy drinkers, were 1.47 (95%CI: 1.27–1.70) in men and 3.97 (95%CI: 2.07–7.61) in women. Similar ORs were observed for beer (males: OR = 3.30; 95%CI: 2.87–3.78; females: OR = 23.1; 95%CI: 12.2–43.6), Port wine (males: OR = 2.21 95%CI: 1.65–2.98; females: OR = 2.85; 95%CI: 0.68–12.1), brandy (males: OR = 3.67 95%CI: 2.98–4.52; females: OR = 13.2; 95%CI: 3.72–46.6) and whisky (males: OR = 3.31; 95%CI: 2.71–4.03; females: OR = 41.4; 95%CI: 18.5–92.5).

**Conclusion:**

This study showed that smokers have a higher intake of alcoholic beverages and a lower consumption of food items rich in fibre, antioxidants, or phytochemicals, which are suspected to have beneficial roles in the prevention of multiple chronic diseases.

## Background

There is evidence favouring the hypothesis that smokers tend to increase their alcohol consumption and to make specific dietary choices [1]. Smoking, poor diet, and physical inactivity tend to cluster, and are all major contributors to the burden of chronic disease [2].

Chronic disease mortality is increasing and is predicted to increase substantially over the next two decades, during which time cardiovascular diseases are expected to remain the leading cause of death [3].

Cigarette smoking increases the risk of all histological types of lung cancer and is also associated with cancer at other locations and with cardiovascular diseases [4]. Each cigarette contains a mixture of carcinogens, tumour promoters, and co-carcinogens. Most tobacco carcinogens require metabolic activation to exert their carcinogenic effects and competing detoxification pathways also exist. The balance between metabolic activation and detoxification may be influenced by micronutrient antioxidants contained in fruit and vegetables [5,6].

The intake of antioxidants such as ascorbic acid protects against the oxidative damage to DNA caused by tobacco smoke [6-9], and ascorbate is depleted by smoking [10,11]. Cigarette smoking is also independently associated with lower circulating concentrations of provitamin A carotenoids [11].

As serum antioxidant nutrient concentration is influenced by smoking, and especially by dietary intake of antioxidant nutrients [12-15], smokers may need higher intakes of fruit and vegetables than do non-smokers. A diet high in fruit and vegetables may be more effective in the reduction of risk for several chronic diseases than large doses of a small number of micronutrient supplements [16].

The health effects of alcohol drinking have been extensively studied. The major diseases and injuries associated with heavy drinking are malignant neoplasms, neuropsychiatric disorders, diabetes mellitus, cardiovascular disorders, cirrhosis of the liver, and intentional and unintentional injuries [17,18]. Alcohol and smoking increase the risk of laryngeal, lung and bladder cancers [18].

Smoking is the result of demographic, economic, and cultural determinants [4], and other factors operating at different phases of the smoking epidemic, such as the politics for controlling tobacco consumption. Most studies evaluating smoking and diet come from Western societies in an advanced phase of the smoking epidemic. Portugal has the highest level of social inequalities in the European Union [19], and appears to be currently in transition from stage 2 to stage 3 of the smoking epidemic. Smoking is more common in men but is still increasing in women, mainly among those who are more educated [20]. Also, in Portugal, access to education appears to be the key determinant of a better food consumption pattern [21], and is an important mediator of the choice of alcoholic beverages. Moreover, the pattern of alcohol consumption is changing in Portugal, with younger people shifting consumption from wine to beer and spirits [22].

These findings suggest the importance of knowing, in depth, how health behaviours are distributed, and how and why they cluster. Such work will play an important role in the design of health promotion programs.

The aim of this study was to compare dietary intake and alcohol consumption, according to smoking status, in a representative sample of the Portuguese general population.

## Methods

### The Portuguese National Health Survey

We analysed data from the third National Health Survey (National Health Observatory, National Institute of Health – Dr. Ricardo Jorge, Ministry of Health), carried out between October 1998 and September 1999. A nationally representative sample of the Portuguese population was obtained using a multi-stage random probability design. Participants (n = 48,606) were selected from 21,808 individual households (collective houses were excluded), distributed in the five regions of mainland Portugal (namely *Norte*, *Centro*, *Lisboa/Vale do Tejo*, *Alentejo *and *Algarve*; these regions are the Portuguese NUTS II subdivisions). This probabilistic sample is representative of the Portuguese population from the Continental area (the *Açores *and *Madeira *archipelagos were not included).

The sample unit was the household, and the basic structure for organization of data collection was based on the population and housing census of 1991. Two strata were defined. First, the *freguesias *(corresponding to counties) were considered, and, within *freguesias*, geographically defined units of approximately 300 household were selected. All subjects living in the sampling unit (household) were surveyed. The survey response rate was 82%.

Trained interviewers conducted face-to-face interviews in each household and obtained information on social and demographic characteristics, lifestyle and health, including smoking, and intakes of selected food and beverages. A quality control was conducted by readministration (by a different interviewer) of the same questionnaire to 10% of the initial sample [23].

The sample used in the present analysis includes all subjects (20,302 women and 17,923 men) older than 19 years, with information on the key variables.

Table 1 summarises the distribution of smoking status by sex, age, and education.

**Table 1:** Demographic characteristics of Portuguese adults, by smoking status.

	**Smoking status**					
	**Total**	**Non-smokers**	**1–9 cig/day**	**10–19 cig/day**	**20 cig/day**	**>20 cig/day**
	**n (%)**	**%**	**%**	**%**	**%**	**%**
**Gender**						
Men	17923 (46.9)	69.3	3.6	6.1	12.4	8.5
Women	20302 (53.1)	91.8	2.2	2.7	2.4	0.8
**Age **(years)						
**Men**						
20–29	3248 (18.1)	59.3	5.0	10.3	17.5	8.0
30–39	2936 (16.4)	51.9	4.9	8.5	20.4	14.3
40–49	3138 (17.5)	62.0	3.5	6.0	14.6	14.0
50–59	2895 (16.2)	72.7	2.8	4.3	11.5	8.7
60–69	2962 (16.5)	81.8	2.9	4.3	6.7	4.3
>69	2744 (15.3)	91.1	2.2	2.8	2.7	1.2
**Women**						
20–29	3025 (14.9)	82.0	5.4	6.1	5.2	1.2
30–39	3164 (15.6)	82.0	4.7	6.5	5.0	1.7
40–49	3422 (16.9)	89.5	2.6	3.1	3.5	1.3
50–59	3337 16.4)	96.0	0.9	1.2	1.2	0.8
60–69	3449 (17.0)	99.0	0.2	0.4	0.3	0.1
>69	3905 (19.2)	99.5	0.3	0.1	0.1	0.1
**Education **(years)						
**Men**						
<4	1514 (8.4)	80.7	2.5	3.3	7.5	5.9
4	6844 (38.2)	72.6	2.7	4.6	11.4	8.6
5–12	6390 (35.7)	58.7	4.7	9.3	16.8	10.5
>12	1367 (7.6)	70.5	4.5	6.4	10.2	8.3
Missing	1808 (10.1)	83.8	3.1	3.1	6.5	3.7
**Women**						
<4	3058 (15.1)	98.7	0.5	0.3	0.4	0.1
4	6371 (31.4)	96.1	1.2	1.2	1.3	0.3
5–12	5582 (27.5)	81.8	4.7	6.2	5.3	2.0
>12	1797 (8.9)	80.7	5.1	6.7	5.4	2.0
Missing	3494 (17.1)	99.7	0.0	0.1	0.1	0.1

### Education

Respondents were asked whether they had obtained further education since leaving school and, if so, the highest qualification completed was noted. Education was recorded as years of education, and was subsequently classified into four levels of education: less than 4 years, 4 years, 5–12 years, and more than 12 years.

### Smoking habits

The questionnaire included detailed questions regarding present and past tobacco consumption: (1) Do you smoke? (daily/occasionally/don't smoke) (2) How many cigarettes do you smoke per day? (3) Since what age do you smoke? (4) Have you ever smoked? (daily/occasionally/never smoked) (5) How many cigarettes did you smoke per day when you smoked? (6) At what age did you start smoking? and (7) At what age did you stop smoking?

For analysis, participants were classified as non-smokers (never smokers, ex-smokers, and those smoking less than one cigarette per day), and smokers (those smoking at least one cigarette per day), grouped into four categories of number of cigarettes smoked per day (1–9, 10–19, 20, and > 20).

### Food and beverage intakes

Respondents were asked 13 questions related to their intake of central food groups and beverages, namely vegetable soup, meat, fish, vegetables, fruit, bread, starchy foods (pasta/rice/potatoes), milk, wine, beer, brandy, whisky, and Port wine, and consumption was recorded as a "yes" (when the respondent indicated the consumption of the food) or "no". Consumption of these food items was determined by asking: "For each of the listed food items please indicate those consumed during the day before the interview" (vegetable soup, meat, fish, vegetables, fruit, bread, and starchy foods [pasta/rice/potatoes]); "during the week before the interview" (wine, beer, brandy, whisky, and Port wine), and "daily" (milk and wine).

The amount of milk and alcoholic beverages consumed in the week before the interview was recorded (number of glasses/day, and glass capacity). The mean consumption of each beverage per day was then computed.

### Statistical analysis

Separate binomial logistic regression models were fitted for males and females, to estimate the magnitude of the association between smoking categories and consumption of particular food groups, adjusting for age and education. Multinomial logistic regression models were fitted separately, by gender, to estimate the association between smoking and drinking categories (non-drinkers, drinkers below the median consumption level, and drinkers above the median consumption level), adjusting for age and education.

Unfortunately, the present Portuguese survey database does not include the variables needed to consider household cluster sampling in the analysis, and no correction was performed for intracluster correlation.

A *P *value of less than 0.05 was considered statistically significant.

## Results

There was a significant decrease in the consumption of vegetable soup, fruit, and vegetables, with increasing number of cigarettes smoked. Table 2 shows the adjusted odds ratios (OR) for food consumption according to smoking categories.

**Table 2:** Odds ratios for food consumption according to smoking status, adjusted for age and education.

	**Men**				**Women**			
	
	**n (%)**	**OR**	**95% CI**	**p trend**	**n (%)**	**OR**	**95% CI**	**p trend**
**Soup**								
Non-smokers	7978 (65.3)	1	[reference]		12106 (65.6)	1	[reference]	
1–9 cig/day	377 (60.1)	0.92	0.77–1.10		230 (51.8)	0.72	0.59–0.87	
10–19 cig/day	578 (53.0)	0.70	0.62–0.80		269 (48.6)	0.64	0.53–0.76	
20 cig/day	1195 (55.0)	0.78	0.71–0.86		216 (44.6)	0.53	0.44–0.64	
>20 cig/day	682 (48.9)	0.60	0.54–0.68	**<0.001**	62 (42.8)	0.46	0.33–0.65	**<0.001**
**Vegetables**								
Non-smokers	10164 (83.2)	1	[reference]		15527 (84.0)	1	[reference]	
1–9 cig/day	501 (80.0)	0.87	0.70–1.09		365 (82.0)	0.80	0.62–1.03	
10–19 cig/day	862 (79.1)	0.79	0.67–0.93		444 (80.6)	0.71	0.57–0.89	
20 cig/day	1688 (77.6)	0.71	0.63–0.80		372 (77.0)	0.57	0.46–0.71	
>20 cig/day	1043 (74.2)	0.56	0.49–0.64	**<0.001**	109 (75.2)	0.47	0.32–0.69	**<0.001**
**Fruit**								
Non-smokers	10929 (89.4)	1	[reference]		16898 (91.4)	1	[reference]	
1–9 cig/day	556 (88.4)	0.99	0.75–1.31		394 (88.5)	0.60	0.44–0.81	
10–19 cig/day	893 (82.1)	0.52	0.43–0.61		492 (88.6)	0.60	0.45–0.79	
20 cig/day	1785 (82.1)	0.53	0.46–0.61		386 (79.4)	0.30	0.23–0.38	
>20 cig/day	1066 (75.7)	0.36	0.31–0.41	**<0.001**	117 (80.1)	0.29	0.19–0.44	**<0.001**
**Bread**								
Non-smokers	11831 (96.6)	1	[reference]		17578 (95.0)	1	[reference]	
1–9 cig/day	607 (96.2)	0.83	0.54–1.27		401 (90.1)	0.61	0.44–0.84	
10–19 cig/day	1047 (96.1)	0.83	0.60–1.15		489 (88.1)	0.50	0.38–0.66	
20 cig/day	2089 (95.6)	0.74	0.58–0.95		424 (87.1)	0.44	0.34–0.59	
>20 cig/day	1359 (96.2)	0.94	0.69–1.28	0.164	123 (84.2)	0.38	0.24–0.60	**<0.001**
**Other starchy**								
Non-smokers	11509 (94.1)	1	[reference]		17040 (92.2)	1	[reference]	
1–9 cig/day	595 (94.4)	1.04	0.70–1.57		413 (92.8)	0.79	0.55–1.15	
10–19 cig/day	1035 (95.1)	1.02	0.75–1.40		509 (92.0)	0.72	0.52–1.00	
20 cig/day	2073 (95.1)	1.00	0.79–1.26		441 (91.1)	0.63	0.46–0.88	
>20 cig/day	1322 (93.8)	0.80	0.62–1.02	0.147	127 (87.6)	0.52	0.31–0.87	**<0.001**
**Fish**								
Non-smokers	6699 (55.1)	1	[reference]		10012 (54.3)	1	[reference]	
1–9 cig/day	330 (52.9)	0.98	0.82–1.16		210 (47.4)	0.76	0.62–0.92	
10–19 cig/day	530 (48.8)	0.84	0.73–0.95		288 (52.5)	0.92	0.78–1.10	
20 cig/day	1121 (51.9)	0.94	0.85–1.04		218 (45.1)	0.69	0.57–0.83	
>20 cig/day	728 (51.9)	0.91	0.81–1.03	**0.030**	69 (47.9)	0.74	0.53–1.03	**<0.001**
**Meat**								
Non-smokers	9672 (79.4)	1	[reference]		13890 (75.2)	1	[reference]	
1–9 cig/day	523 (83.7)	1.04	0.81–1.32		376 (84.7)	1.03	0.79–1.35	
10–19 cig/day	924 (84.9)	1.06	0.88–1.28		473 (85.5)	1.08	0.84–1.39	
20 cig/day	1853 (85.2)	0.98	0.85–1.12		415 (85.9)	1.11	0.85–1.44	
>20 cig/day	1162 (82.9)	0.83	0.71–0.97	0.055	117 (80.7)	0.87	0.57–1.34	0.769

When heavy smokers were compared with non-smokers, the OR favouring soup consumption was 0.60 (95% Confidence Interval [95%CI]: 0.54–0.68) in males and 0.46 (95% CI: 0.33–0.65) in females. Similar ORs were observed for vegetables (males: OR = 0.56, 95%CI: 0.49–0.64; females: OR = 0.47, 95%CI: 0.32–0.69) and fruit (males: OR = 0.36, 95%CI: 0.31–0.41; females: OR = 0.29, 95%CI: 0.19–0.44).

With bread and other starchy foods (potatoes, pasta and rice), only women smokers showed a statistically significant consumption trend. The consumption of bread and other starchy food was significantly lower in females who smoked more than 20 cigarettes/day, compared to non-smokers (bread: OR = 0.38, 95%CI: 0.24–0.60; starchy foods: OR = 0.52, 95% CI: 0.31–0.87). As shown in Table 2, the ORs of fish consumption through the categories of smoking, although statistically significant in both sexes, did not show a linear trend.

No significant association between smoking and meat consumption during the day before the interview was observed in either gender.

Tables 3 and 4 show the adjusted ORs for beverage consumption, according to smoking categories.

**Table 3:** Odds ratios for beverage consumption according to smoking status, adjusted for age and education (men).

	**Non-drinkers**	**Drinkers (< median) vs. Non-drinkers**				**Drinkers (> median) vs. Non-drinkers**			
	**n (%)**	**n (%)**	**OR**	**95%CI**	**p trend**	**n (%)**	**OR**	**95%CI**	**p trend**

**Milk**									
Non-smokers	3273 (68.2)	4102 (68.9)	1	[reference]		3484 (67.1)	1	[reference]	
1–9 cig/day	144 (3.0)	237 (4.0)	1.27	1.02–1.57		197 (3.8)	1.17	0.93–1.47	
10–19 cig/day	259 (5.4)	384 (6.4)	1.13	0.95–1.33		396 (7.6)	1.28	1.08–1.51	
20 cig/day	637 (13.3)	780 (13.1)	0.99	0.88–1.11		682 (13.1)	1.00	0.89–1.14	
>20 cig/day	483 (10.1)	451 (7.6)	0.89	0.67–0.89	**0.003**	436 (8.4)	0.92	0.80–1.07	0.610
**Wine**									
Non-smokers	4084 (68.9)	3342 (69.5)	1	[reference]		3325 (66.1)	1	[reference]	
1–9 cig/day	198 (3.3)	204 (4.2)	1.47	1.19–1.82		173 (3.4)	1.40	1.12–1.77	
10–19 cig/day	431 (7.3)	311 (6.5)	1.08	0.92–1.26		292 (5.8)	1.16	0.98–1.38	
20 cig/day	790 (13.3)	573 (11.9)	1.00	0.88–1.13		700 (13.9)	1.28	1.13–1.45	
>20 cig/day	422 (7.1)	379 (7.9)	1.01	0.87–1.18	0.781	542 (10.8)	1.47	1.27–1.70	**<0.001**
**Beer**									
Non-smokers	7432 (75.7)	1664 (65.0)	1	[reference]		1499 (48.1)	1	[reference]	
1–9 cig/day	325 (3.3)	122 (4.8)	1.45	1.17–1.80		113 (3.6)	1.37	1.09–1.72	
10–19 cig/day	530 (5.4)	201 (7.9)	1.46	1.22–1.74		286 (9.2)	2.08	1.78–2.45	
20 cig/day	971 (9.9)	362 (14.1)	1.34	1.17–1.53		689 (22.1)	2.47	2.19–2.77	
>20 cig/day	563 (5.7)	212 (8.3)	1.28	1.08–1.52	**<0.001**	528 (17.0)	3.30	2.87–3.78	**<0.001**
**Brandy**									
Non-smokers	9931 (69.7)	474 (62.0)	1	[reference]		353 (46.8)	1	[reference]	
1–9 cig/day	523 (3.7)	27 (3.5)	1.15	0.77–1.72		22 (2.9)	1.32	0.84–2.05	
10–19 cig/day	927 (6.5)	58 (7.6)	1.44	1.08–1.92		49 (6.5)	1.75	1.28–2.39	
20 cig/day	1774 (12.4)	120 (15.7)	1.38	1.11–1.71		173 (22.9)	2.75	2.26–3.35	
>20 cig/day	1094 (7.7)	85 (11.1)	1.44	1.13–1.84	**<0.001**	158 (20.9)	3.67	2.98–4.52	**<0.001**
**Whisky**									
Non-smokers	9862 (70.1)	477 (59.8)	1	[reference]		377 (46.8)	1	[reference]	
1–9 cig/day	495 (3.5)	42 (5.3)	1.60	1.15–2.24		38 (4.7)	1.81	1.28–2.58	
10–19 cig/day	895 (6.4)	64 (8.0)	1.35	1.02–1.77		65 (8.1)	1.72	1.30–2.27	
20 cig/day	1758 (12.5)	125 (15.7)	1.31	1.06–1.62		153 (19.0)	2.00	1.64–2.44	
>20 cig/day	1065 (7.6)	90 (11.3)	1.42	1.12–1.80	**<0.001**	172 (21.4)	3.31	2.71–4.03	**<0.001**
**Port wine**									
Non-smokers	10227 (68.9)	286 (68.4)	1	[reference]		205 (50.9)	1	[reference]	
1–9 cig/day	537 (3.6)	14 (3.4)	0.86	0.50–1.48		21 (5.2)	1.80	1.14–2.86	
10–19 cig/day	977 (6.6)	18 (4.3)	0.60	0.37–0.98		37 (9.2)	1.74	1.21–2.50	
20 cig/day	1928 (13.0)	49 (11.7)	0.82	0.60–1.12		77 (19.1)	1.77	1.34–2.33	
>20 cig/day	1223 (8.2)	51 (12.2)	1.31	0.96–1.79	0.905	63 (15.6)	2.21	1.65–2.98	**<0.001**

**Table 4:** Odds ratios for beverage consumption according to smoking status, adjusted for age and education (women).

	**Non-drinkers**	**Drinkers (< median) vs. Non-drinkers**				**Drinkers (> median) vs. Non-drinkers**			
	
	**n (%)**	**n (%)**	**OR**	**95%CI**	**p trend**	**n (%)**	**OR**	**95%CI**	**p trend**
**Milk**									
Non-smokers	3731 (91.7)	6122 (90.3)	1	[reference]		5255 (89.3)	1	[reference]	
1–9 cig/day	88 (2.2)	191 (2.8)	1.16	0.89–1.50		167 (2.8)	1.15	0.88–1.50	
10–19 cig/day	96 (2.4)	213 (3.1)	1.16	0.90–1.48		243 (4.1)	1.49	1.17–1.91	
20 cig/day	115 (2.8)	196 (2.9)	0.90	0.71–1.14		173 (2.9)	0.90	0.70–1.15	
>20 cig/day	40 (1.0)	58 (0.9)	0.72	0.48–1.09	0.322	49 (0.8)	0.66	0.43–1.01	0.798
**Wine**									
Non-smokers	11293 (90.9)	3337 (88.5)	1	[reference]		479 (89.2)	1	[reference]	
1–9 cig/day	319 (2.6)	118 (3.1)	1.45	1.16–1.81		9 (1.7)	1.01	0.51–2.00	
10–19 cig/day	386 (3.1)	144 (3.8)	1.44	1.18–1.76		20 (3.7)	1.93	1.19–3.11	
20 cig/day	330 (2.7)	134 (3.6)	1.54	1.24–1.90		18 (3.4)	2.00	1.21–3.30	
>20 cig/day	95 (0.8)	38 (1.0)	1.34	0.91–1.97	**<0.001**	11 (2.0)	3.97	2.07–7.61	**<0.001**
**Beer**									
Non-smokers	14536 (91.5)	525 (72.3)	1	[reference]		58 (47.9)	1	[reference]	
1–9 cig/day	384 (2.4)	48 (6.6)	2.45	1.78–3.37		14 (11.6)	6.52	3.54–12.0	
10–19 cig/day	479 (3.0)	59 (8.1)	2.30	1.72–3.08		10 (8.3)	3.63	1.81–7.28	
20 cig/day	382 (2.4)	73 (10.1)	3.67	2.79–4.81		25 (20.7)	11.6	7.01–19.1	
>20 cig/day	108 (0.7)	21 (2.9)	3.67	2.26–5.95	**<0.001**	14 (11.6)	23.1	12.2–43.6	**<0.001**
Brandy *									
Non-smokers	15117 (90.4)	32 (68.1)	1	[reference]					
1–9 cig/day	444 (2.7)	3 (6.4)	4.76	1.38–16.4					
10–19 cig/day	549 (3.3)	3 (6.4)	4.05	1.16–14.2					
20 cig/day	476 (2.9)	6 (12.8)	8.42	3.27–21.7					
>20 cig/day	144 (0.9)	3 (6.4)	13.2	3.72–46.6	**<0.001**				
**Whisky**									
Non-smokers	15037 (90.8)	83 (57.6)	1	[reference]		23 (43.4)	1	[reference]	
1–9 cig/day	431 (2.6)	11 (7.6)	3.33	1.73–6.39		4 (7.6)	4.83	1.61–14.4	
10–19 cig/day	526 (3.2)	18 (12.5)	4.17	2.43–7.15		7 (13.2)	6.71	2.74–16.4	
20 cig/day	452 (2.7)	20 (13.9)	5.45	3.24–9.15		8 (15.1)	9.08	3.87–21.3	
>20 cig/day	121 (0.7)	12 (8.3)	10.8	5.62–20.9	**<0.001**	11 (20.8)	41.4	18.5–92.5	**<0.001**
**Port wine**									
Non-smokers	14836 (90.6)	207 (77,5)	1	[reference]		57 (80,3)	1	[reference]	
1–9 cig/day	426 (2.6)	17 (6.4)	2.13	1.28–3.57		3 (4.2)	1.45	0.44–4.72	
10–19 cig/day	535 (3.3)	13 (4.9)	1.21	0.68–2.16		4 (5.6)	1.48	0.52–4.19	
20 cig/day	453 (2.8)	23 (8.6)	2.60	1.66–4.09		5 (7.0)	2.23	0.87–5.70	
>20 cig/day	135 (0.8)	7 (2.6)	2.42	1.11–5.28	**<0.001**	2 (2.8)	2.85	0.68–12.1	**0.030**

* the results are presented for non-drinkers and drinkers of any amount of brandy.

Male heavy smokers, compared to non-smokers, presented lower odds favouring milk consumption (OR = 0.89, 95%CI: 0.67–0.89), when milk drinkers below the median consumption level were compared with non-drinkers. No such association was found for women.

When heavy smokers were compared with non-smokers, the ORs favouring wine drinking, among heavy drinkers, were 1.47 (95%CI: 1.27–1.70) in men and 3.97 (95%CI: 2.07–7.61) in women. Similar ORs were observed for beer (males: OR = 3.30; 95%CI: 2.87–3.78; females: OR = 23.1; 95%CI: 12.2–43.6), Port wine (males: OR = 2.21 95%CI: 1.65–2.98; females: OR = 2.85; 95%CI: 0.68–12.1), brandy (males: OR = 3.67 95%CI: 2.98–4.52; females: OR = 13.2; 95%CI: 3.72–46.6) and whisky (males: OR = 3.31; 95%CI: 2.71–4.03; females: OR = 41.4; 95%CI: 18.5–92.5).

## Discussion

Our study showed that smoking is associated with less healthy dietary choices and higher alcohol consumption in the Portuguese population. Heavy smokers consumed significantly less vegetable soup, vegetables, and fruit compared to non-smokers, independent of age and education. Smoking was associated with higher intakes of all alcoholic beverages analysed.

Our investigation is based on a large representative sample of the Portuguese population, but some limitations of this study need to be addressed. The methods for dietary assessment employed generic classifications of food groups, rather than specific varieties or species. Quantitative measures were generally not used, and only a limited number of food items were considered. As a result, we could not estimate the quantity or specific composition of food consumed. The reporting of dietary habits is known to be influenced by personal characteristics, and the association between smoking and the patterns of food and beverage consumption might be attributed to differential reporting in smokers and non-smokers, unless increased intakes of other items could be documented, to show maintenance of the energy balance. Unfortunately, the questionnaire included a very restricted number of food items, and we did not have data on total energy consumed. The literature indicates, however, that while smokers eat less of some food groups (the more healthy foods, such as vegetables and fruit) [1,24], they tend to report increased intakes of other food groups (such as chips, fatty meats, and sugar) [25,26], that were not included in this survey. We must also bear in mind that alcoholic beverages (contributing to energy intake) are consumed much more frequently by smokers.

Another limitation of the Portuguese National Health Survey is that a proportion of the data on education level is missing (the data were lost for 17% of women and 10% of men). This fact decreased the proportions of participants in the final regression models. For that reason we have included, in Table 1, a breakdown (by smoking habits) of subjects whose education information is missing. As their smoking pattern was similar to that presented by subjects with less than 4 years of education, the overall prevalence of smoking was probably overestimated.

The impossibility of accounting for the effects of cluster sampling in our analysis contributed to an underestimation of the variances. The statistical power of our study is higher than it would be if the analysis was conducted considering intracluster correlation (the design effect increases with the size of the clusters and with the intracluster correlation coefficient [27]). Our conclusions cannot, however, be invalidated by limitations of the analysis. Given the magnitude of the associations observed, and the dose-response relations seen, our conclusions are robust. It is unlikely that the associations shown in this study would disappear if the design effects resulting from the complex sampling procedure were considered in the analysis. Male and female data were analysed separately. Usually, therefore, only one participant with the same gender was sampled in most households, and the average size of the clusters is much lower than 2. We may assume a design effect of 2 as a highly conservative estimate of what would be obtained with analysis procedures allowing for intracluster correlation. This is true even when assuming that the correlation between subjects within the same cluster can be conservatively assumed to be close to 1 (near the maximum). We emphasize that when the ORs for soup, fruit and vegetable consumptions in the two highest categories of cigarette smoking are considered, the standard errors would have to rise more than 3.5-fold, on average, to make the associations statistically non-significant. The ORs favouring the consumption of higher amounts of most alcoholic beverages, when the two highest categories of cigarette smoking were compared with non-smokers, and the dose-response relationships are even more robust than these abovementioned associations.

An additional issue that needs to be considered is that OR estimates may change depending on the method used to perform the analysis of surveys with complex sampling procedures, but the effects of different approaches are difficult to predict. An empirical comparison [28] of different methods for analysis of cluster randomised trials (standard logistic regression, standard logistic regression with robust standard errors, generalized estimating equation, random-effects logistic regression, Bayesian random-effects regression) showed differences not larger than 13% between the logs of ORs estimated through standard logistic regression, and those using any of the other methods. Our estimates easily accommodate differences of this magnitude, towards the null, without compromising the conclusions.

In our analysis, we classified individuals who smoke less than one cigarettes per day (2.4%) as non-smokers. Ex-smokers (14.4%) were also included in the non-smoking category because we believe they would be more similar to non-smokers than to current smokers. However, we cannot exclude instances of misclassification.

Several studies have reported that compared to smokers, non-smokers are more likely to consume fruit and vegetables [1,15,24-26,29-42], items rich in fibre, antioxidants, and phytochemicals. Our results are also consistent with those obtained when nutrients, instead of food items, were analysed. Several studies have found that non-smokers had higher intakes of vitamins, antioxidants, and fibre [1,9,35,43-46], which are found in vegetables and fruit.

There is consistent evidence for a protective effect of vegetable and fruit consumption against cancer of several types [47-50], and against cardiovascular disease [48,51]. Thus, smokers reported food intake choices that may further aggravate their smoking-related risk of cancer and cardiovascular diseases. A significant inverse association was also found between intake of fibre, fruit, and vegetables, and mortality [52].

In agreement with Whichelow et al. [30] and Tonstad et al. [43], we found that women who smoked more consumed significantly less starchy foods (bread, potatoes, pasta and rice). Whichelow et al. [30], Margetts and Jackson [26] in the UK, and Osler [40] in Denmark, showed that non-smokers consumed more "brown" bread. The type of bread consumed was not recorded in the survey data used here, so our results show only that non-smokers were more likely to consume bread.

Non-smokers were also found to be more likely to eat fish. A similar effect was found in Norwegian men [39].

In some populations, smokers were shown to consume more meat and meat dishes [7,26,39,40], but other studies recorded a higher intake of poultry by non-smokers [30,53]. We found no significant association between meat intake and smoking status, although we were unable to specify the meat types consumed.

We observed a significant association between smoking and the consumption of wine, beer, brandy, whisky and Port wine, in both genders, confirming a well-known relationship between smoking and alcohol consumption [1,30,37,38,40,41,44,46,53-59].

In Portugal, smokers have a less healthy diet, and higher alcohol intake. These findings are consistent with data indicating that patterns of health behaviour tend to cluster [60,61]. This implies that a global programme on health promotion, addressing lifestyle factors as a block, is required. Such a comprehensive approach should include strategies to control smoking and alcohol consumption, to improve diet, and to enhance physical activity.

These results need to be considered when designing research or intervention studies on cancer and cardiovascular diseases, which are related to smoking, diet, and alcohol consumption.

## Conclusion

This study of the general population in Portugal showed that compared to non-smokers, smokers have a higher intake of alcoholic beverages and a lower consumption of food items rich in fibre, antioxidants, or phytochemicals. A programme aimed at addressing lifestyle factors as a block must be considered for the prevention of chronic diseases.

## Competing interests

The author(s) declare that they have no competing interests.

## Authors' contributions

PP, NL and HB designed the study. PP, A-C S and NL did the statistical analysis. PP, NL and HB wrote the paper. All authors read and approved the final version of the paper.

## Pre-publication history

The pre-publication history for this paper can be accessed here:



## References

[B1] Dallongeville J, Marécaux N, Fruchart JC, Amouyel P (1998). Cigarette smoking is associated with unhealthy patterns of nutrient intake: a Meta-analysis. J Nutr.

[B2] Yach D, Leeder SR, Bell J, Kistnasamy B (2005). Global chronic diseases. Science.

[B3] Yach D, Hawkes C, CL G, Hofman KJ (2004). The Global Burden of Chronic Diseases. Overcoming Impediments to Prevention and Control. JAMA.

[B4] Ezzati M, Lopez AD (2003). Estimates of global mortality attributable to smoking in 2000. Lancet.

[B5] Wogan GN, Hecht SS, Felton JS, Conney AH, Loeb LA (2004). Environmental and chemical carcinogenesis. Semin Cancer Biol.

[B6] Frei B (2004). Efficacy of dietary antioxidants to prevent oxidative damage and inhibit chronic disease.. J Nutr.

[B7] Ma J, Hampl JS, Betts NM (2000). Antioxidant intakes and smoking status: data from the Continuing Survey of Food Intakes by Individuals. Am J Clin Nutr.

[B8] Chen K, Suh J, Carr AC, Morrow JD, Zeind J, Frei B (2000). Vitamin C suppresses oxidative lipid damage in vivo, even in the presence of iron overload.. Am J Physiol Endocrinol Metab.

[B9] Trobs M, Renner T, Scherer G, Heller WD, Geiss HC, Wolfram G, Haas GM, Schandt P (2002). Nutrition, antioxidants, and risk factor profile of nonsmokers, passive smokers and smokers of the Prevention Education Program (PEP) in Nuremberg, Germany. Prev Med.

[B10] Lykkesfeldt J, Christen S, Wallock LM, Chang HH, Jacob RA, Ames BN (2000). Ascorbate is depleted by smoking and repleted by moderate supplementation: a study in male smokers and nonsmokers with matched dietary antioxidant intakes. Am J Clin Nutr.

[B11] Alberg AJ (2002). The influence of cigarette smoking on circulating concentrations of antioxidant micronutrients. Toxicology.

[B12] Galan P, Viteri FE, Bertrais S, Czernichow S, Faure H, Arnaud J, Ruffieux D, Chenal S, Arnault N, Favier A, Roussel AM, Hercberg S (2005). Serum concentrations of [beta]-carotene, vitamins C and E, zinc and selenium are influenced by sex, age, diet, smoking status, alcohol consumption and corpulence in a general French adult population. Eur J Clin Nutr.

[B13] Dietrich M, Block G, Norkus EP, Hudes M, Traber MG, Cross CE, Packer L (2003). Smoking and exposure to environmental tobacco smoke drecrease some plasma antioxidants and increase Y-tocopherol in vivo after adjustment for dietary antioxidant intakes. Am J Clin Nutr.

[B14] Faure H, Preziosi P, Roussel AM, Bertrais S, Galan P, Hercberg S, Favier A (2006). Factors influencing blood concentration of retinol, [alpha]-tocopherol, vitamin C, and [beta]-carotene in the French participants of the SU.VI.MAX trial. Eur J Clin Nutr.

[B15] Marangon K, Herbeth B, Lecomte E, Paul-Dauphin A, Grolier P, Chancerelle Y, Artur Y, Siest G (1998). Diet, antioxidant status, and smoking habits in French men. Am J Clin Nutr.

[B16] Woodside JV, McCall D, McGartland C, Young IS (2005). Micronutrients: dietary intake v. supplement use. Proc Nutr Soc.

[B17] Room R, Babor T, Rehm J (2005). Alcohol and public health. Lancet.

[B18] Bagnardi V, Blangiardo M, La Vecchia C, Corrao G (2001). Alcohol consumption and the risk of cancer: a meta-analysis.. Alcohol Res Health.

[B19] Barreto A, Preto CV, Rosa MJV, Lobo MC, Chitas P (2000). A situação social em Portugal 1960-1999: Indicadores sociais em Portugal e na União Europeia [The social situation in Portugal 1960-1999: Social indicators in Portugal and the European Union].

[B20] Santos AC, Barros H (2004). Smoking patterns in a community sample of Portuguese adults, 1999-2000. Prev Med.

[B21] Moreira PA, Padrao PD (2004). Educational and economic determinants of food intake in Portuguese adults: a cross-sectional survey.. BMC Public Health.

[B22] Marques-Vidal P, Dias C (2005). Trends and Determinants of Alcohol Consumption in Portugal: Results From the National Health Surveys 1995 to 1996 and 1998 to 1999. [Miscellaneous Article]. Alcohol Clin Exp Res.

[B23] Ministério da Saúde. Instituto Nacional de Saúde Dr. Ricardo Jorge (2001). Inquérito Nacional de Saúde-Nota Metodológica.

[B24] Palaniappan U, Starkey LJ, O'Loughlin J, Gray-Donald K (2001). Fruit and vegetable consumption is lower and saturated fat intake is higher among Canadians reporting smoking. J Nutr.

[B25] Hebert JR, Kabat GC (1990). Differences in dietary intake associated with smoking status. Eur J Clin Nutr.

[B26] Margetts BM, Jackson AA (1993). Interations between people's diet and their smoking habits:the dietary and nutritional survey of British adults. BMJ.

[B27] Donner A, Birkett N, Buck C (1981). Randomization by cluster: sample size requirements and analysis. Am J Epidemiol.

[B28] Peters TJ, Richards SH, Bankhead CR, Ades AE, Sterne JAC (2003). Comparison of methods for analysing cluster randomized trials: an example involving a factorial design. Int J Epidemiol.

[B29] Woo J, Ho SC, Sham A, Leung SSF, Lam TH, Janus ED (2001). Dietary habit of smokers in a Chinese population. Int J Food Sci Nutr.

[B30] Whichelow MJ, Erzinclioglu SW, Cox BD (1991). A comparison of the diets of non-smokers and smokers. Br J Addict.

[B31] Osler M, Tjonneland A, Suntum M, Thomsen BL, Stripp C, Gronbaek M, Overvad K (2002). Does the association between smoking status and selected healthy foods depend on gender? A population-based study of 54 417 middle-aged Danes. Eur J Clin Nutr.

[B32] Billson H, Pryer JA, Nichols R (1999). Variation in fruit and vegetable consumption among adults in Britain. An analysis from the dietary and nutritional survey of British adults. Eur J Clin Nutr.

[B33] Pollard J, Greenwood D, Kirk S, Cade J (2001). Lifestyle factors affecting fruit and vegetable consumption in the UK Women's Cohort Study. Appetite.

[B34] Wilson DB, Nietert PJ (2002). Patterns of fruit, vegetable, and milk consumption among smoking and nonsmoking female teens. Am J Prev Med.

[B35] Koh WP, Yuan JM, Sun CL, Lee HP, Yu MC (2005). Middle-Aged and Older Chinese Men and Women in Singapore Who Smoke Have Less Healthy Diets and Lifestyles than Nonsmokers. J Nutr.

[B36] Wilson DB, Smith BN, Speizer IS, Bean MK, KS M, Uguy LS, Fries EA (2005). Differences in food intake and exercise by smoking status in adolescents. Prev Med.

[B37] McPhillips JB, Eaton CB, Gans KM, Derby CA, Lasater TM, McKenney JL, Carleton RA (1994). Dietary differences in smokers and nonsmokers from two southeastern New England communities. J Am Diet Assoc.

[B38] Morabia A, Wynder EL (1990). Dietary habits of smokers, people who never smoked, and exsmokers. Am J Clin Nutr.

[B39] Oshaug A, Bjonnes CH, Bugge KH, Trygg KU (1996). Tobacco smoking, an independent determinant for unhealthy diet? A cross-sectional study of Norwegian workers on platforms in the North Sea. Eur J Public Health.

[B40] Osler M (1998). The food intake of smokers and nonsmokers: the role of partner's smoking behavior. Prev Med.

[B41] Morabia A, Curtin F, Bernstein MS (1999). Effects of smoking and smoking cessation on dietary habits of a Swiss urban population. Eur J Clin Nutr.

[B42] La Vecchia C, Negri E, Franceschi S, Parazzini F, Decarli A (1992). Differences in dietary intake with smoking, alcohol, and education. Nutr Cancer.

[B43] Tonstad S, Gorbitz C, Sivertsen M, Ose L (1999). Under-reporting of dietary intake by smoking and non-smoking subjects counselled for hypercholesterolaemia. J Intern Med.

[B44] Woodward M, Bolton-Smith C, Tunstall-Pedoe H (1994). Deficient health knowledge, diet, and other lifestyles in smokers: is a multifactorial approach required?. Prev Med.

[B45] Mammas I, Bertsias G, Linardakis M, Moschandreas J, Kafatos A (2004). Nutrient intake and food consumption among medical students in Greece assessed during a Clinical Nutrition course. International Journal of Food Sciences and Nutrition.

[B46] Fehily AM, Phillips KM, Yarnell WG (1984). Diet, smoking, social class, and body mass index in the Caerphilly Heart Disease Study. Am J Clin Nutr.

[B47] Steinmetz KA, Potter JD (1996). Vegetable, fruit, and cancer prevention: a review. J Am Diet Assoc.

[B48] Hercberg S, Galan P, Preziosi P, Alfarez MJ, Vasquez C (1998). The potential role of antioxidant vitamins in preventing cardiovascular diseases and cancer. Nutrition.

[B49] Holick CN, Michaud DS, Stolzenberg-Solomon R, Mayne ST, Pietinen P, Taylor PR, Virtamo J, Albanes D (2002). Dietary Carotenoids, Serum {beta}-Carotene, and Retinol and Risk of Lung Cancer in the Alpha-Tocopherol, Beta-Carotene Cohort Study. Am J Epidemiol.

[B50] Lunet N, Lacerda-Vieira A, Barros H (2005). Fruit and Vegetables Consumption and Gastric Cancer: A Systematic Review and Meta-Analysis of Cohort Studies. Nutr Cancer.

[B51] Ness AR, Powles JW (1997). Fruit and vegetables, and cardiovascular disease: a review.. Int J Epidemiol.

[B52] Knoops KTB, Groot LC, Fidanza F, Alberti-Fidanza A, Kromhout D, van Staveren WA (2006). Comparison of three different dietary scores in relation to 10-year mortality in elderly European subjects: the HALE project. Eur J Clin Nutr.

[B53] Majem LS, Vinas BR, Barba LR, Ramon JM, Lloveras G (2001). Relación del consumo de alimentos y nutrientes con el hábito tabáquico. Med Clin (Barc).

[B54] Rust P, Lehner P, Elmadfa I (2001). Relationship between dietary intake, antioxidant status and smoking habits in female Austrian smokers. Eur J Clin Nutr.

[B55] Laaksonen M, Lahelma E, Prattala R (2002). Associations among health-related behaviours: sociodemographic variation in Finland. Soz- Praventivmed.

[B56] Ramos E, Barros H (1998). Diet and smoking in Spain and Portugal: a comment. Eur J Public Health.

[B57] Burke V, Milligan AK, Beilin LJ, Spencer DM, Balde E, Gracey MP (1997). Clustering of Health-Related Behaviors among 18-Year-Old Australians. Prev Med.

[B58] Fisher M, Gordon T (1985). The relation of drinking and smoking habits to diet: the Lipid Research Clinics Prevalence Study. Am J Clin Nutr.

[B59] Wetzels JJL, Kremers SPJ, Vitória PD, de Vries H (2003). Tha alcohol-tobacco relationship: a prospective study among adolescents in six European countries. Addiction.

[B60] Berrigan D, Dodd K, Troiano RP, Krebs-Smith SM, Barbash RB (2003). Patterns of health behavior in U.S. adults. Prev Med.

[B61] Galan I, Rodriguez-Artalejo F, Diez-Ganan L, Tobias A, Zorrilla B, Gandarillas A (2006). Clustering of behavioural risk factors and compliance with clinical preventive recommendations in Spain. Prev Med.

